# Deep learning‐based classification and structure name standardization for organ at risk and target delineations in prostate cancer radiotherapy

**DOI:** 10.1002/acm2.13446

**Published:** 2021-10-08

**Authors:** Christian Jamtheim Gustafsson, Michael Lempart, Johan Swärd, Emilia Persson, Tufve Nyholm, Camilla Thellenberg Karlsson, Jonas Scherman

**Affiliations:** ^1^ Department of Hematology Oncology and Radiation Physics Skåne University Hospital Lund Sweden; ^2^ Department of Translational Sciences Medical Radiation Physics Lund University Malmö Sweden; ^3^ Centre for Mathematical Sciences Mathematical Statistics Lund University Lund Sweden; ^4^ Department of Radiation Sciences Radiation Physics Umeå University Umeå Sweden; ^5^ Department of Radiation Sciences Oncology Umeå University Umeå Sweden

**Keywords:** classification, deep learning, machine learning, radiotherapy, structure

## Abstract

Radiotherapy (RT) datasets can suffer from variations in annotation of organ at risk (OAR) and target structures. Annotation standards exist, but their description for prostate targets is limited. This restricts the use of such data for supervised machine learning purposes as it requires properly annotated data. The aim of this work was to develop a modality independent deep learning (DL) model for automatic classification and annotation of prostate RT DICOM structures.

Delineated prostate organs at risk (OAR), support‐ and target structures (gross tumor volume [GTV]/clinical target volume [CTV]/planning target volume [PTV]), along with or without separate vesicles and/or lymph nodes, were extracted as binary masks from 1854 patients. An image modality independent 2D InceptionResNetV2 classification network was trained with varying amounts of training data using four image input channels. Channel 1–3 consisted of orthogonal 2D projections from each individual binary structure. The fourth channel contained a summation of the other available binary structure masks. Structure classification performance was assessed in independent CT (*n* = 200 pat) and magnetic resonance imaging (MRI) (*n* = 40 pat) test datasets and an external CT (*n* = 99 pat) dataset from another clinic.

A weighted classification accuracy of 99.4% was achieved during training. The unweighted classification accuracy and the weighted average F1 score among different structures in the CT test dataset were 98.8% and 98.4% and 98.6% and 98.5% for the MRI test dataset, respectively. The external CT dataset yielded the corresponding results 98.4% and 98.7% when analyzed for trained structures only, and results from the full dataset yielded 79.6% and 75.2%. Most misclassifications in the external CT dataset occurred due to multiple CTVs and PTVs being fused together, which was not included in the training data.

Our proposed DL‐based method for automated renaming and standardization of prostate radiotherapy annotations shows great potential. Clinic specific contouring standards however need to be represented in the training data for successful use. Source code is available at https://github.com/jamtheim/DicomRTStructRenamerPublic

## 1 INTRODUCTION

The use of artificial intelligence (AI) and machine learning for radiation oncology enable automation and optimization of the clinical workflow. Deep learning (DL) is a machine learning technique which has gained a lot of attention in the last years due to leaps in image classification performance^1^ and segmentation.^2^ Several reviews on the use of AI and DL for radiotherapy (RT) applications have been written.^3^, ^4^, ^5^, ^6^, ^7^, ^8^, ^9^, ^10^, ^11^ The most popular applications have focused on automatic structure delineation, automatic treatment planning, and synthetic computed tomography (CT) generation.^3^, ^12^

Delineation of organs at risk (OAR) and targets has traditionally been performed in a manual, time‐consuming manner which is associated with intra‐ and inter‐observer variability.^13^, ^14^, ^15^ DL‐based segmentation can improve consistency and efficiency,^16^, ^17^, ^18^, ^19^ can produce segmentations with clinically acceptable quality,^20^, ^21^ and has outperformed previous atlas‐based solutions.^16^ Training of robust and generalizable supervised DL models relies on correctly annotated ground truth training data (RT structure annotation labels) and sufficient clinical heterogeneity.^3^ A standardized nomenclature for the RT structures is essential for all types of automatic data extraction to facilitate model development or data analysis.^11^, ^22^, ^23^, ^24^ Collection of high quality annotated clinical data from one or multiple clinics can be cumbersome if an RT structure name standardization is non‐existing.^24^, ^25^

To exemplify, “FemoralHead_R,” “caput dx,” “caput dx1,” and “avoid dx,” were different names for the right femoral head structure found in our clinical data. Similar examples for other structures have been presented.^22^, ^23^, ^26^, ^27^, ^28^ To mitigate and avoid such annotation problems, a comprehensive naming schema has been presented by the American Association of Physicists in Medicine (AAPM) Task Group 263 (TG‐263).^24^ Swedish University Hospitals aim to follow the naming standard defined in Santanam et al^25^ and ICRU^29^ and has been documented in detail.^30^, ^31^

An excellent example of inferior auto segmentation performance as a function of increasing noise in annotation labels is provided by Yu et al.^32^ Data cleaning, renaming, and quality control (QC) are therefore required for creation of high quality datasets aimed for machine learning.^33^ This can be heavily time consuming,^34^ and it is estimated that a dominant part of the research time is spent on data cleaning.^35^ Automation of this process would therefore be highly beneficial, and several previous methods have been presented. The use of text‐based logic to capture and correct variations in structure names has shown good results.^30^, ^34^, ^36^ However, it can be difficult to maintain and support such logic, especially if adaptations to data from multiple institutions or languages are needed, or if the naming convention changes over time.^23^, ^25^, ^26^, ^27^, ^34^ An extensive work using DL natural language processing on text labels to possible mediate such problems was performed by Syed et al^23^ for prostate and lung. However, the use of text‐based methods only accounts for semantic differences in the label names and blindly assumes the delineation data of the RT structure to be representative and correct.

Further, existing naming conventions for prostate targets do not contain information whether multiple anatomical targets are included within the same target, i.e. is it only prostate gland or gland including seminal vesicles or/and lymph nodes.^24^, ^25^, ^31^ To conclude, existing naming conventions do not offer data granularity to a level needed for the above purposes, and text‐based approaches might be limited in its potential. This highly motives the development of image‐based methods for RT structure classification.

Previous image‐based attempts using machine learning methods for lung and heart have been presented.^22^, ^28^ Studies using DL image classification using convolutional neural networks for head and neck^26^, ^27^, ^37^ and prostate^26^, ^27^ have also been presented. However, recent studies have focused on OAR classification only^26^, ^37^ and ignored the target structures, where classification has been pointed out as a challenging task.^26^ Further, CT images were required as model input in most of the existing image‐based methods,^22^, ^26^, ^28^, ^37^ thereby excluding data from novel magnetic resonance imaging (MRI) only treatment planning techniques.^38^, ^39^, ^40^

By extending upon previous studies, the aim of this work was to focus on developing, evaluating, and verifying an open‐source image modality independent supervised DL model for automated renaming and standardization of clinical prostate cancer RT structures. The objectives of the model were to classify several OAR, support structures, a gross tumor volume (GTV) target structure, the clinical target volume (CTV) and planning target volume (PTV) for multiple different targets, both pre‐ and post‐operative. QC mechanisms for the structure classification were implemented, and the effects of varying the amount of training data and model input were investigated.

## 2 METHODS

### 2.1 Description of datasets

CT and MRI images with DICOM structure delineation data for retrospective prostate cancer patients were automatically extracted from the clinical treatment planning system (Eclipse v.15.6, VARIAN, Palo Alto, CA, USA) at the Department of Hematology, Oncology and Radiation Physics at Skåne University Hospital, Lund, Sweden, using Eclipse scripting application programming interface. Extracted patients with conventional CT had received external beam RT treatment between 2016‐01‐01 and 2020‐08‐19 using volumetric modulated arc therapy (VMAT). No selection of specific prostate targets was performed and thereby included prostatectomized patients and patients with prostate gland, with or without involvement of the vesicles and/or iliac lymph nodes. Structure delineations were based on Gay et al,^41^ and delineation of the prostate bed was performed according to Poortmans et al.^42^ Structure templates were used during initial creation of the structures, and most of them had separate CTVs and PTVs.

Patients contoured on MRI images originated from a previous prostate MRI only treatment study,^39^ where MRI data were converted to synthetic CT data for the purpose of VMAT treatment planning to the prostate gland only. The dataset contained two CTV structures per patient where one of the CTVs had 1 mm extra margin (excluding cranio‐caudal extension). All CT and MRI patient target delineations were performed in VARIAN Eclipse by oncologists and organ at risk (OAR) delineation was performed by oncologists and dosimetrists.

Additional inclusion criteria used for patient selection were the number of RT fractions ≥6 with a fractional dose of >1.8 Gy. Actions were taken to assert data integrity for CT, MRI, and RT structures. This resulted in a CT‐based cohort of 2054 subjects with mean age of 70.4 ± 6.2 (1 SD) (41–87 years) (*n* = 2054). The MRI‐based cohort was based on 40 subjects with a mean age of 71.1 ± 5.6 (1 SD) (49–81 years) (*n* = 40).

An additional dataset from the RT clinic at Umeå University Hospital, Umeå, Sweden was available through a common collaboration. The dataset contained DICOM CT images and RT structure delineation data from 99 prostate cancer patients (one patient had two scans) who received external beam RT treatment between 2011 and 2017. Target volumes were represented by prostate gland, vesicles, and lymph nodes. Unlike the data from Skåne University Hospital, the CTV and PTV target volumes commonly included multiple structures in a variety of boolean combinations, for example, prostate gland + vesicles + lymph nodes or vesicles + lymph nodes. Another difference was the addition of prostate gland boost volumes, multiple other OARs, and a support structure. These differences constituted in a total of 11 additional structure types, when compared to the datasets from Skåne University Hospital. The mean age of all subjects was 69.3 (54–83) years (*n* = 99). The treatment planning system used to create the data was Oncentra External Beam (v.4.5.2, Elekta, Stockholm, Sweden). Throughout this study, the authors have aimed to fulfil the checklist for AI in medical imaging described in Mongan et al.^43^ The study was funded by Skåne University Hospital, Lund, Sweden and the Swedish government innovation agency VINNOVA, supporting the ASSIST project with Grant No. 2019–04735. Ethical approval was defined within the ASSIST project framework (Application 2020‐02009, Swedish Ethical Review Authority).

### 2.2 Creation of datasets

Two hundred subjects (10%) were randomly selected from the Skåne University Hospital CT cohort of 2054 patients, further referred to as the CT test dataset, leaving 1854 subjects to train the model on, further referred to as the training dataset. The 40 patients with MRI are referred to as the MRI test dataset. The Umeå clinic dataset was accompanied with a verified ground truth and was used as an external, second test dataset, referred to as the Umeå test dataset. The three (CT, MRI, and Umeå) test datasets were never used in model training or optimization.

The data contents of the delineated structures in the training dataset, described in Table SA1, were verified by a licensed medical physicist. Two hundred ninety mislabeled structures were detected and recorded together with corrections in a separate comma‐separated value (CSV) text file, referred to as the class label correction file. A verified ground truth was thereby established for the training data. The structure content of the CT and MRI test dataset was manually analyzed by a licensed medical physicist after model inference. The structure content is presented in Table SA1.

The clinical structure names, description for the training and test data, and the class label definition used in this study are shown in Table SA1. Structure names beginning with the text “Tuning,” “Help,” “X,” “Y,” “Z,” “Dose,” or “Match” (any letter case) were identified using text‐based label rules and are in this work referred to as optimization structures. Such structures are used to shape the dose distribution, optimize, and individualize the treatment plan.

### 2.3 Data preprocessing

The training dataset was constructed by converting each delineated RT structure for each subject to a 3D binary mask NiFTI file^44^ (background = 0, foreground = 1) using the Python package dcmrtstruct2nii (v.1.0.19).^45^ The input to dcmrtstruct2nii required both CT files and an RT structure set. CT data were therefore extracted for this purpose; however it was not used during any model training.

**FIGURE 1:**
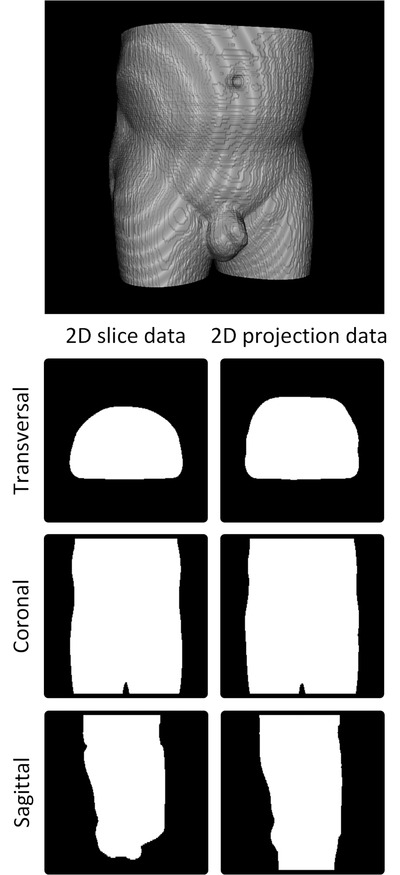
Volume rendering of binary 3D body mask data from a male pelvis. 2D binary transversal, coronal, and sagittal slices from the 3D binary mask are shown at the body mask center of mass point (left column) together with calculated 2D projection data (right column).

**FIGURE 2:**
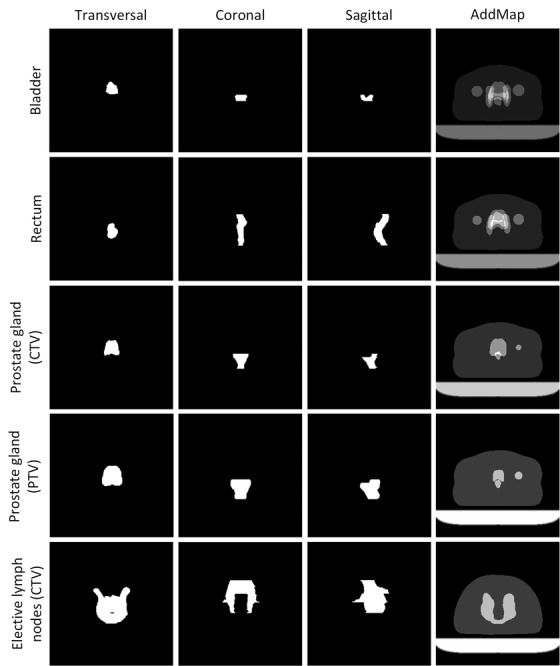
Input images to the model as projection data for transversal, coronal, and sagittal views together with corresponding AddMap for described organs at risks and targets. AddMap was created from a weighted sum over all other radiotherapy (RT) structures in the center of mass transversal slice of the organ of interest. As an example, the prostate gland CTV structure is shown in row 3 where the corresponding PTV structure can be seen in the prostate gland CTV AddMap. Due to the added margin, the planning target volume (PTV) is larger than the clinical target volume (CTV). The reverse relationship for the AddMap holds for the prostate gland PTV structure in row 4. These relationships were used to supply the neural network with intra‐ and inter‐structure spatial and geometric information. Further on, an elective lymph node CTV structure is shown in row 5 where the corresponding PTV can be seen in the AddMap with a “U” shape. The CTV structure does not show the “U” shape seen in the AddMap PTV due to the construction of projection data, populating voxels within the “U” shape. Images originate from the same patient and have been rotated and/or flipped for improved viewing. Individual gray scales were applied to AddMap in the figure to visualize each organ, actual pixel values were assigned in the same way for all individual structure AddMap.

The DL model was designed to use four different 2D images as the input data, supplied as four different image channels. Three images were defined by a transversal, coronal, and sagittal 2D projection of each individual RT structure 3D binary mask volume, referred to as projection data, that is, each RT structure in each subject produced three projection images. The projection data pixel values were determined by summing up the voxel's values (0 or 1) that fell in the way of parallel rays traced from the viewpoint to the projection plane, and the sum was truncated to a maximum value of 1, generating a 2D binary mask. The projection data were equivalent to a 2D shadow from a 3D object, where the area within the shadow boundary was assigned the value 1 and all other values 0 (Figures 1 and 2). This allowed some of the 3D structure information to be condensed into three 2D images. The fourth image consisted of a weighted sum of all other available RT structure binary masks from the subject and is further referred to as the AddMap. Optimization structures were ignored in the above image generation processes.

The AddMap was created to provide the neural network with spatial information regarding the surrounding, that is, location of other structures and the body contour. Further, it was hypothesized that it could facilitate differentiation between CTV and PTV structures. The AddMap was created as a 2D image and saved as a NiFTI file. The slice location was determined at the center of mass for the structure of interest. The weights for other binary masks in the summation were 0.1 for the BODY and 0.2 for all other structures, not to be confused with class label weights in Table SA1. Signal summation in the AddMap was truncated to the maximum pixel value of 1 (Figure 2). Creation of AddMap is similar to the method used by Rozario et al.^27^ but extended to contain signal truncation. Examples of projection data and AddMap for multiple structures can be seen in Figure 2.

To enable faster data access during model training, the NiFTI structure data were read, down sampled to 256 × 256 using nearest neighbor interpolation and stored in an HDF5 database.^46^ Data were stored together with the class label according to Table SA1 using an anonymized subject ID. Any deviation in the training data RT structure names, recorded in the class label correction file (see “creation of datasets”), were accounted for, and the class label was corrected. To minimize central processing unit (CPU) strain and maximize graphics processing unit (GPU) training utilization, projection and label data were saved as int8. The AddMap was saved as float32. The structure volume from the original data was calculated before resampling and stored together with its class label in an additional HDF5 database. The volume measure was only used for QC purposes, and not as model input.

### 2.4 Model training, optimization, and inference

#### 2.4.1 Model training

An InceptionResNetV2 model^47^ was selected from Keras in the Tensorflow framework (v.2.2.0, Google).^48^ The InceptionResNetV2 model has 164 layers and utilizes inception modules,^49^ a rectified linear unit (ReLU) activation function, and residual connections.^50^ The neural network layer weights were randomly initialized as prescribed by Keras default, and the model was trained using supervised classification, utilizing a softmax classifier in the last layer. A weighted categorical cross entropy loss function was used during model training to accommodate class label imbalances, originating from differences in number of available structures for each class in the training data (Table SA1). The class weights were calculated as a balanced distribution using the Python package Scikit‐learn,^51^ that is, the inverse of the label frequency distribution was used as weights (Table SA1). Adaptive moment estimation (Adam) was chosen as an optimizer with a learning rate of 0.001.^52^ A learning rate decay factor of 0.2 was applied if no change in validation accuracy was detected within 10 epochs. The output of the network consisted of a probability given for each defined class, and the largest probability defined the determined class. The model was trained using 10‐fold cross‐validation (90% training, 10% validation data), where data were separated on subject level.

Dataset generation, calculations, and model training were performed on a system equipped with an AMD Ryzen Threadripper2 2990 WX 64 thread CPU, 128 GB RAM and two NVIDIA TITAN RTX GPUs running Ubuntu 18.04.3 with CUDA version 10.1 and NVIDIA driver version 440.82. Source code and software documentation used to preprocess data or to perform model training and inference is available on GitHub at https://github.com/jamtheim/DicomRTStructRenamerPublic.

#### 2.4.2 Investigation and optimization of model parameters and input data

Investigated parameters for model training were batch size, number of epochs, learning rate, and number of input image channels. Model optimization was guided by maximizing the class weighted (by class frequency) classification accuracy in the validation dataset. This metric was reported through Tensorboard in Tensorflow which also provided guidance to avoid model overfitting for all experiments.^48^ A text file, containing the number of failed structures per class was created with evaluation results after 100 epochs to identify difficult classes. The final model parameters were set to batch size 72, 100 epochs and the previously defined learning rate schedule. To investigate the model performance with respect to training data volume, different models were trained with final model parameters using 10‐fold cross‐validation using 10%, 25%, 50%, 75%, and 100% of the available training data. Furthermore, the impact of including or excluding AddMap images as a model input was investigated by calculating the error rate in prostate gland CTV and PTV classification (class label 11 and 12 in Table SA1) on validation data using 100% of the training data. For the above experiments, data were randomly selected on subject level, and the weighted classification accuracy on validation data was evaluated. Note that the cross‐validation splits were different between different fractions of training data but were the same within experiments for a specific fraction. To further validate that the final model focused on relevant features in the input data and to provide model explainability, channel‐wise guided Grad‐Cam^53^ saliency maps were calculated.^54^ The maps were Z‐score normalized, and data within ±2 standard deviations from the mean were selected, masked with the binary structure, rescaled, and overlaid on the input data. The saliency maps showed the most influential pixel areas for determining the class label.

#### 2.4.3 Test dataset inference and QC

Data from the test datasets were loaded directly from its DICOM format, and the same preprocessing pipeline was used as for the training data. In summary, structures were converted to projection data, and AddMap was created to define a four‐channel input to the neural network. The volume of each structure was recorded but not used as model input. The optimization structures were ignored using name text logic mentioned in section “creation of datasets,” and empty structures were also ignored.

**FIGURE 3:**
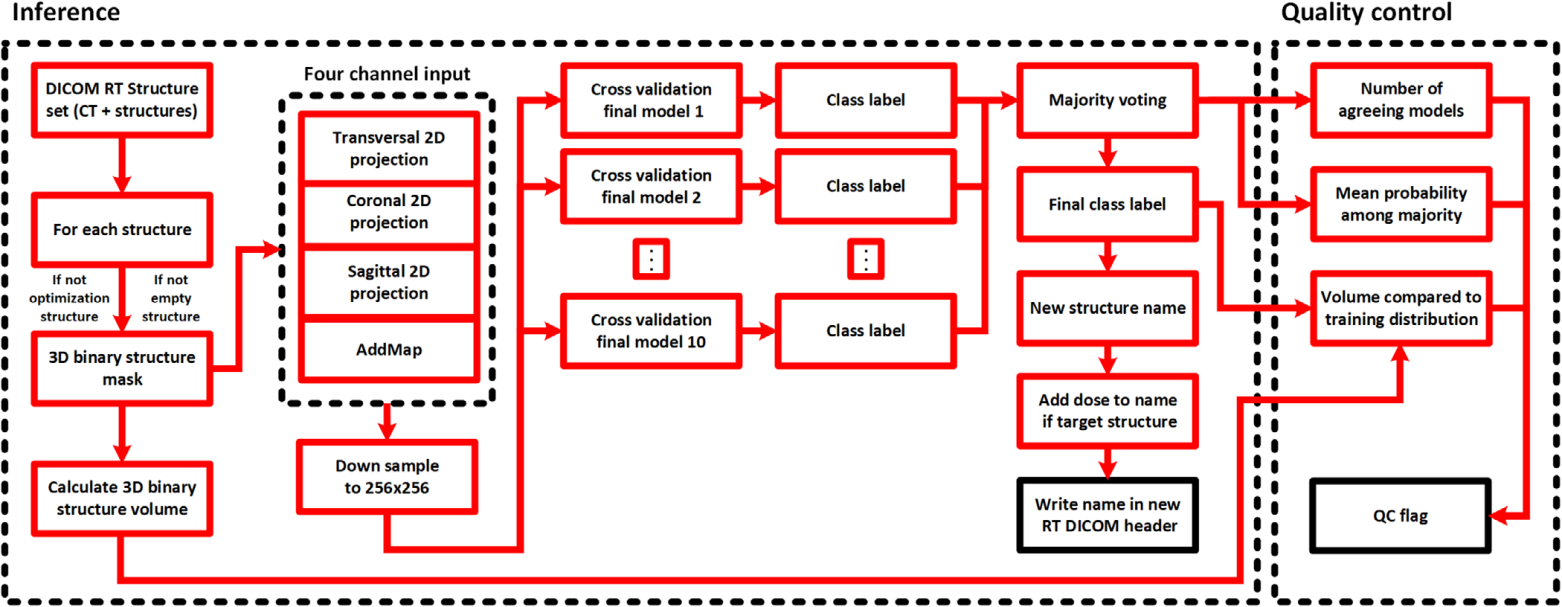
A block diagram of the method workflow. The inference compartment contains the necessary steps for extracting and preprocessing the DICOM radiotherapy (RT) structure data. Data are then provided to the cross‐validation models, and a majority vote among the models is used to decide the final structure label and name. A new structure name is written to a copy of the RT DICOM structure header (black box). The quality control compartment contains the quality control mechanisms, providing a quality control flag for each structure after inference (black box)

Inference on the test dataset using the CPU was performed using the final model for each cross‐validation, that is, every structure was exposed to 10 inference models. A majority vote among the class label output from the 10 models was performed, and the final class label decision was established (Figure 3, “Inference”). The original structure name and final class label were recorded in a results CSV text file.

QC of the final class label was performed using three different methods, and if any method detected QC deviations, a QC flag was raised and recorded in a CSV text file, and no further QC mechanism was executed. Firstly, the structure volume was confirmed to be within the 1st and 99th percentile of the training data volume distribution for the same class label. Secondly, the number of agreeing models was confirmed to be no less than six models. This was performed to ensure that a substantial number of models gave the same output. A third check was performed to confirm if the mean probability of the established class label among the majority voting models was below 0.7. A low probability score could indicate that the model was provided with an input on which it had not been trained (Figure 3, “QC”).

As a final step, the established final class label was translated to a descriptive structure name defined in this study (Table SA1), and the structure name was changed in a copy of the original RT structure DICOM file using the Python package Pydicom.^55^ Hence, after model inference, a new RT structure DICOM file with edited structure names was written, but no image data were altered. Prior to the above‐mentioned process, prescribed dose values to target structures were extracted from the original label name and added to new structure name. Data integrity and validity of the produced RT structure files were confirmed for one subject by importing it and the corresponding CT image volume to VARIAN Eclipse.

### 2.5 Model performance evaluation

The performance of the final model was assessed on all test datasets (CT, MRI, and Umeå) by a licensed medical physicist manually comparing the result from the inference on each structure with respect to the geometric content of the ground truth. For all test datasets, an additional result analysis was performed, where certain structures were not accounted for, as their class was not included in the training of the final model. This will further be referred to as using uncleaned and cleaned data, respectively, and only impacted the result analysis.

To provide a detailed view of the model performance, the classification metrics precision, recall, and F1 score for each class label were assessed in each test datasets together with global unweighted average (macro avg) and weighted average (weighted avg) performance measures using the Python package Scikit‐learn. Weighting was performed with respect to the class label frequency given in the “number of structures” column of respective result table. The classification precision, recall, F1 score, and accuracy of the QC method were also assessed for each dataset. Definitions of these metrics are found in supplementary material.

## 3 RESULTS

### 3.1 Model training and data input dependency

Training of each fold in the cross‐validation for the final model with 100% of the training data took 3 h for 100 epochs. A mean weighted classification accuracy of 99.4% was achieved on the validation data for the 10‐fold cross‐validation. Results from using 10%, 25%, 50%, and 75% of the training data while including or excluding AddMap to the model input are presented in Table 1. No model overfitting was observed for any experiment. The prostate gland CTV and PTV classification mean error (± 1 std) rate from the cross‐validations was calculated to 1.6% ± 1.1% and 1.7% ± 0.7%, respectively, when AddMap was included in the model input and 6.5% ± 2.0% and 4.9% ± 1.3%, respectively, when AddMap was excluded. An example of channel wise Guided Grad‐Cam saliency maps overlaid on input data from different structures can be seen in Figure A1 in supplementary material, visually demonstrating the use of AddMap.

**TABLE 1:** Weighted classification accuracy (mean ± 1 population standard deviation (range)) on validation data for 10‐fold cross‐validations as a function of different fractions of available training data while including or excluding the AddMap as model input

	10% training data	25% training data	50% training data	75% training data	100% training data
Weighted classification accuracy (excluding AddMap)	0.954 ± 0.014 (0.924–0.965)	0.969 ± 0.0062 (0.960–0.979)	0.977 ± 0.0060 (0.963–0.985)	0.983 ± 0.0028 (0.979–0.988)	0.984 ± 0.0024 (0.979–0.988)
Weighted classification accuracy (including AddMap)	0.982 ± 0.011 (0.965–1.00)	0.983 ± 0.0047 (0.976–0.991)	0.991 ± 0.0035 (0.985–0.996)	0.992 ± 0.0033 (0.987–0.997)	0.994 ± 0.0015 (0.990–0.996)

### 3.2 Test dataset evaluation

Classification performance together with QC assessment is presented in separate sections for the CT, MRI, and Umeå test dataset.

### 3.3 CT test dataset

**FIGURE 4:**
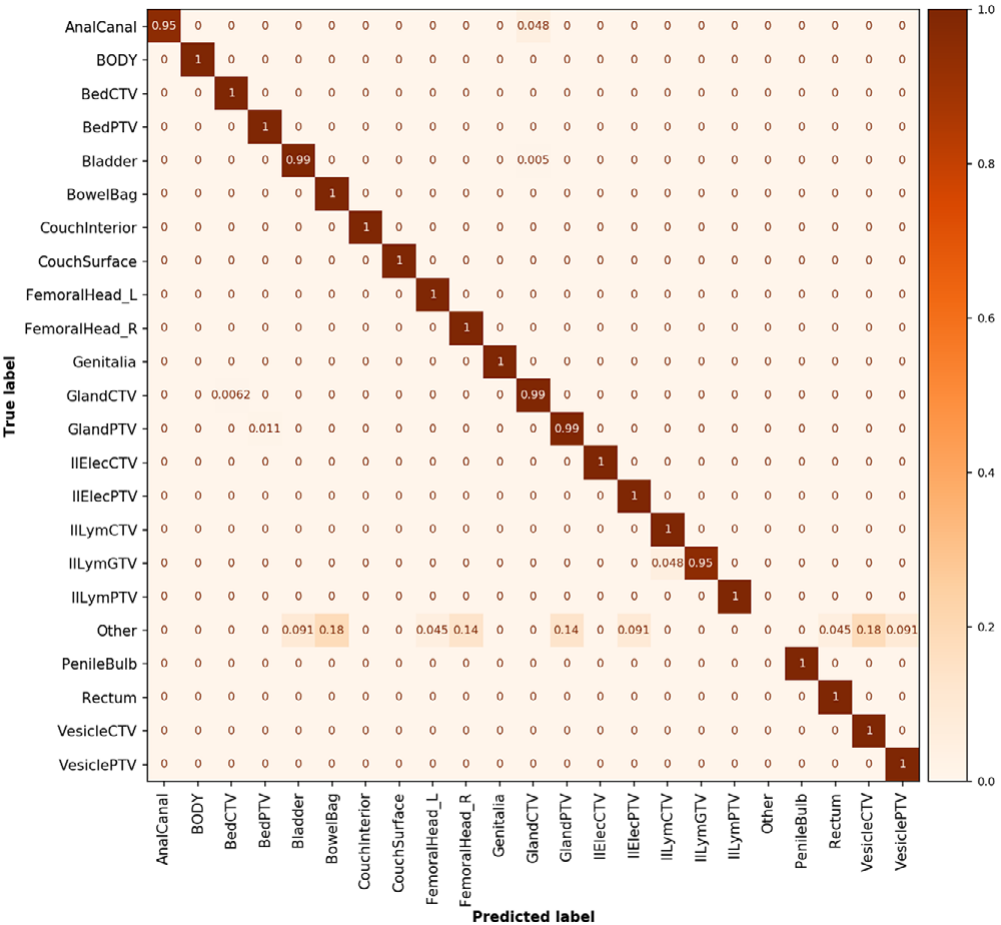
Normalized confusion matrix for the whole CT test dataset. A perfect score generates only diagonal values with value 1. Note that the “Other” class label is not populated for the predictions as the class was not accounted for in the training, but added in the analysis for clarity. Number of objects in each class is given in Table SA2.

A total of 989 optimization structures were automatically ignored using the text‐based label rules defined in section “creation of datasets,” while 2391 structures were included in the analysis of the CT test dataset. Twenty‐eight structures were misclassified, and their predicted class label can be seen in the confusion matrix (Figure 4). Thirteen of the 28 failing structures were identified as mislabeled optimization structures (missing name prefix, see section “creation of datasets”) and were thereby not ignored as intended. Nine structures contained data on which the network had not been trained on. This resulted in only six misclassified structures due to the model and the class labels used to train it. During analysis, the ground truth class “other” was assigned to both the mislabeled and to the network unknown structures (Figure 4 and Table SA2).

This yielded an unweighted classification accuracy of 98.8% and a weighted average F1 score of 98.4% for the whole dataset (uncleaned data). After removal of data in the “other” class label, the analysis yielded an unweighted accuracy of 99.8% and a weighted average F1 score of 99.8% (cleaned data, six of 2369 failed). Detailed individual class label performance metrics for uncleaned and cleaned data are presented in Tables SA2 and SA3, respectively. The QC method precision, recall, F1 score, and accuracy were assessed for the whole CT test dataset and were 14%, 32%, 20%, and 97%, respectively. A total of 64 QC flags were issued, 59 were triggered on the structure volume and five on the majority vote.

### 3.4 MRI test dataset

**FIGURE 5:**
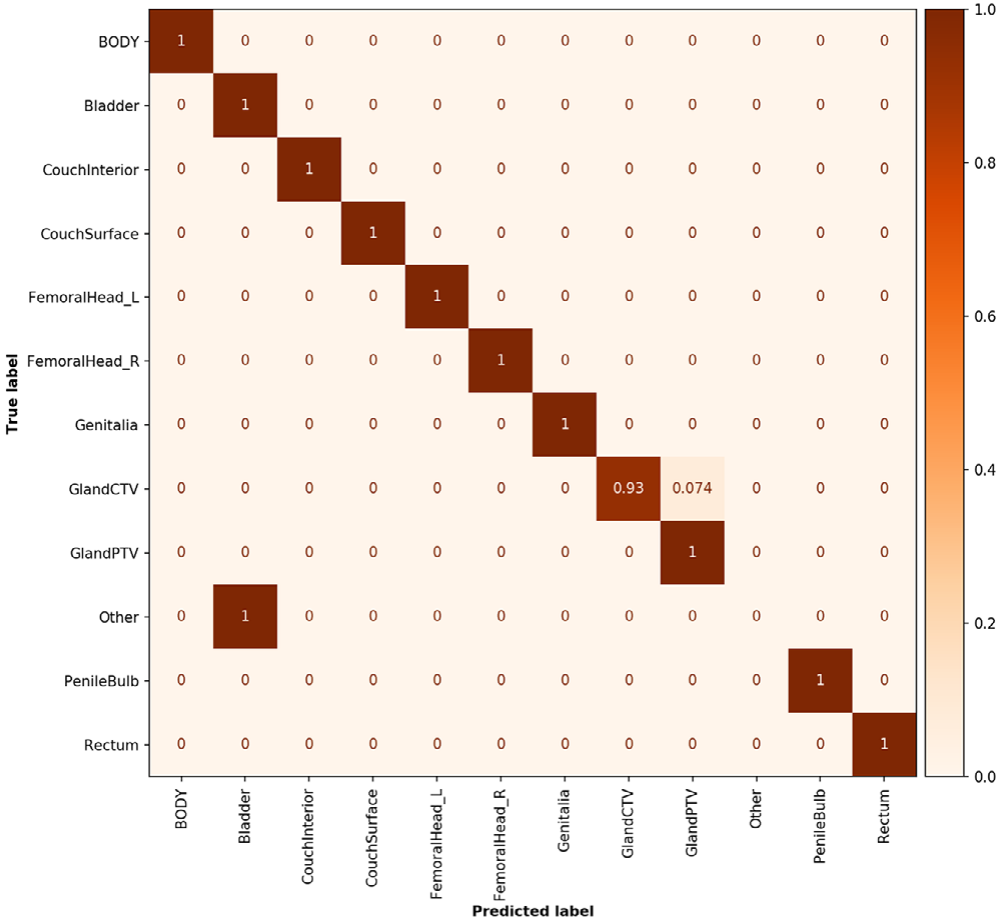
Normalized confusion matrix for the whole magnetic resonance imaging (MRI) test dataset. Note that the “Other” class label is not populated for the predictions as the class was not accounted for in the training but added in the analysis for clarity. Number of objects in each class is given in Table SA4 where the high number of GlandCTV compared to the other class labels was due to the existence of two clinical target volumes (CTVs) per subject in the MRI dataset.

A total of 211 optimization structures were automatically ignored using the text‐based label rules defined in section "creation of datasets,” while 482 structures were included in the analysis of the MRI test data. Seven structures were misclassified (Figure 5). One of the seven failing structures was identified as a mislabeled optimization structure, and thereby not ignored as intended. A ground truth class “other” was assigned to these structures in the analysis (Figure 5 and Table SA4). This resulted in six misclassified structures due to the model and the class labels used to train it.

This yielded an unweighted classification accuracy of 98.6% and a weighted average F1 score of 98.5% for the whole dataset (uncleaned data). After removal of data in the “other” class label, the analysis yielded an unweighted accuracy of 98.8% and a weighted average F1 score of 98.8% (cleaned data, six of 481 failed). Detailed individual class label performance metrics for uncleaned and cleaned data are presented in Table SA4 and Table SA5, respectively.

A total of 127 QC flags were issued, 126 were triggered on the structure volumes for Body, CouchSurface and CouchInterior, due to a smaller MR scan volume compared to the CT training data. One QC flag was issued on the majority vote. The QC method precision, recall, F1, and accuracy score were assessed for the whole MRI dataset and were 30%, 43%, 35% and 98%, respectively (where the volume error was only accounted one time for Body, CouchSurface and CouchInterior).

### 3.5 Umeå test dataset

**FIGURE 6:**
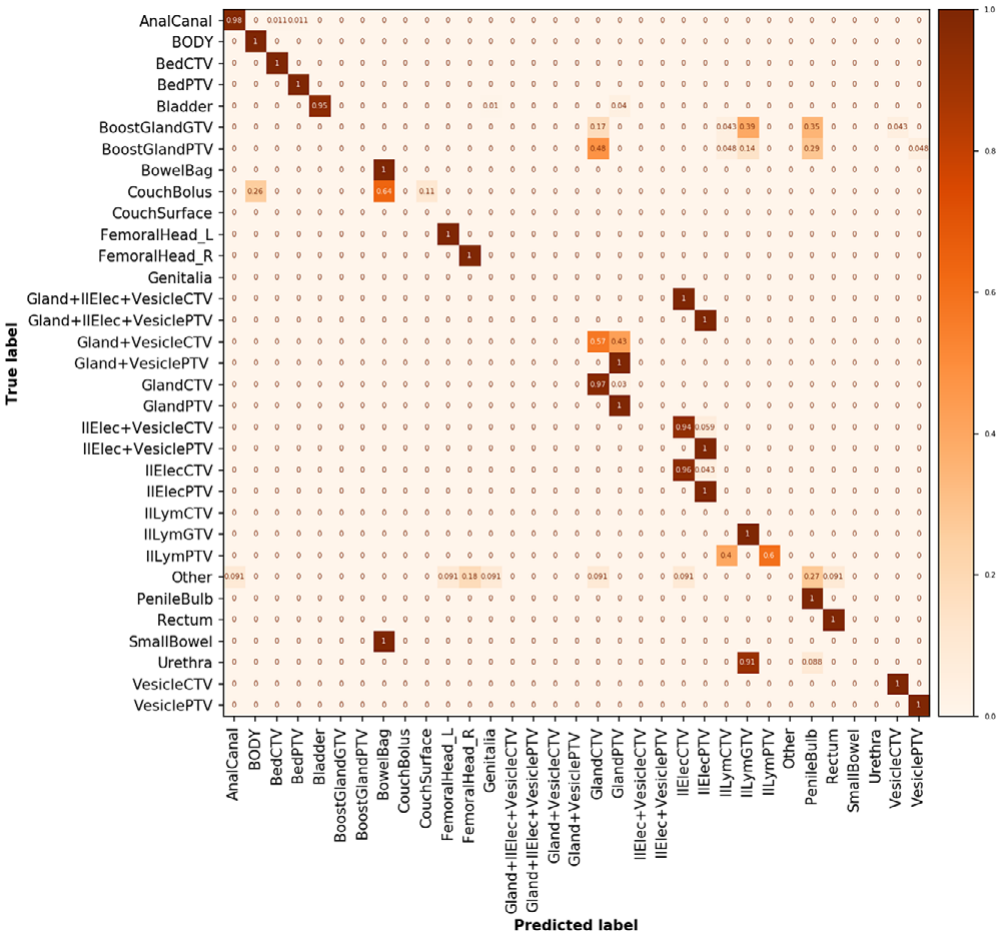
Normalized confusion matrix for the whole Umeå test dataset. Note that the 11 additional class labels are not populated for the predictions as the class was not accounted for in the training, but added in the analysis for clarity. Number of objects in each class is given in Table SA6

A total of 63 optimization structures were automatically ignored using the text‐based label rules defined in the section “creation of datasets,” while 1186 structures were included in the analysis of the Umeå test data. Two hundred forty‐two structures were misclassified (Figure 6), and 227 of these failed, as the model had not been trained on the 11 new class labels, these data contained (Table SA6). Examples of these new class labels are visualized in Figure A2 in supplementary material. Fifteen structures were misclassified despite being supported by the model.

**FIGURE 7:**
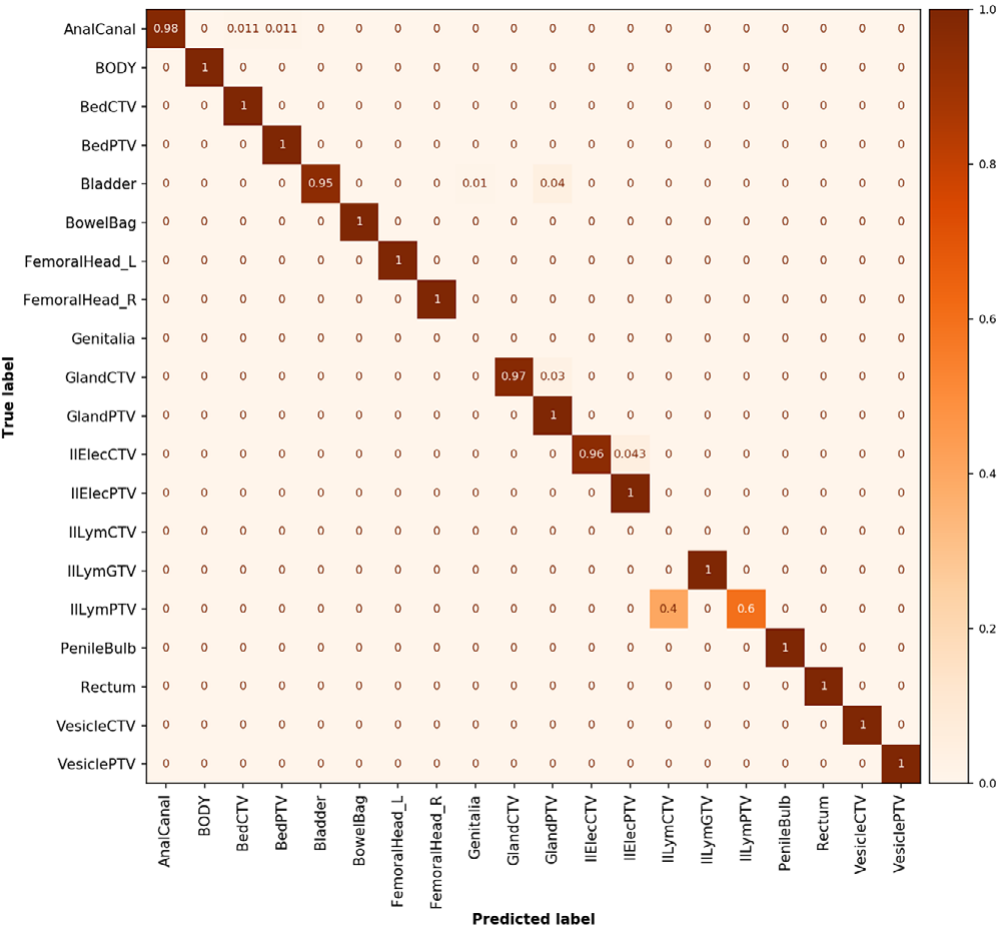
Normalized confusion matrix for the cleaned Umeå test dataset. Number of objects in each class is given in Table SA7

This yielded an unweighted classification accuracy of 79.6% and a weighted average F1 score of 75.2% for the whole dataset (uncleaned data). After the removal of data in the unsupported and “other” class labels, the analysis yielded an unweighted accuracy of 98.4% and a weighted average F1 score of 98.7% (Figure 7, cleaned data, 15 of 959 failed). Detailed individual class label performance metrics for uncleaned and cleaned data are presented in Tables SA6 and SA7, respectively.

A total of 139 QC flags were issued, 108 were triggered on the structure volume. Thirty QC flags were issued on the majority vote and one on the classification acceptance probability. The QC method precision, recall, F1, and accuracy score were assessed for the whole Umeå dataset and were 69%, 38%, 49%, and 84%, respectively.

## 4 DISCUSSION

In this study, an open‐source DL model for automatic prostate cancer RT OAR and target structure classification and renaming was developed. Datasets in DICOM format were converted and supplied as the model input, and RT structures were automatically renamed to a custom‐defined name standard. The model was trained and optimized on a large clinical subject cohort containing a large variety of clinical diagnosis and subject data. RT optimization structures were ignored using text‐based logic, and the model required only binary masks as the input, making the model image modality independent. The impact of varying the training data volume and model input was investigated, and the model showed excellent results on both internal clinical uncleaned CT and MRI test data and cleaned clinical external CT data from another RT clinic (Umeå test data). QC mechanisms were developed and evaluated to notify the user when the proposed method was uncertain regarding its output.

The trained model reached a mean weighted accuracy of 99.4% for the validation data when using 100% of the training data. The investigation regarding impact of available training data demonstrated that classification score decreased, while cross‐validation standard deviation increased with the use of less training data (Table 1). However, a weighted validation classification accuracy of 98.2% was achieved with only 10% of available training data, corresponding to 205 subjects, proving that the model can be trained with a limited dataset.

The input to the model was defined as three orthogonal 2D images, derived from a binary 3D mask as volume projection data, and one 2D image containing a weighted sum of all the other structures (AddMap). A one percentage point increase in weighted classification accuracy was achieved by adding the AddMap to the model input when using 100% training data (Table 1). The positive classification accuracy contribution of AddMap seemed to be of larger importance for smaller data volumes (Table 1, comparing 10% and 100% training data). This demonstrated the added value of supplying the neural network with intra‐ and inter‐structure spatial and geometric information, enabling the network to learn valuable features when the amount of training data is limited. The value of including AddMap was further demonstrated by the results regarding the classification of prostate gland CTV and PTV structures, which showed a 5 and 3 percentage point improvement, respectively (see Figure 2 and Figure SA1 for CTV and PTV example).

Unweighted classification accuracy of 98.8%, 98.6%, 79.6% and corresponding weighted F1 scores of 98.4%, 98.5%, 75.2% was achieved on the uncleaned CT, MRI, and Umeå test datasets, respectively. On the cleaned datasets, an unweighted classification accuracy and a weighted F1 score of 99.8%, 99.8% (CT), 98.8%, 98.8% (MRI), and 98.4%, 98.7% (Umeå) was achieved.

QC of the model output was identified as a future implementation in Syed et al.,^23^ and we approached this by developing multiple different QC mechanisms. The precision, recall, F1 score, and accuracy of the QC for the CT test data were 0.14, 0.32, 0.20, 0.97, for the MRI test data 0.30, 0.43, 0.35, 0.98, and for the Umeå test data 0.69, 0.38, 0.49, 0.84. Low values for precision and recall indicate that the suggested QC methods need to be improved. Aside from the inferior QC performance, the excellent classification results demonstrated a classification model with high performance for targets, OAR, and support structures in multimodal or multicenter prostate RT datasets.

Previous work aiming to classify structures has mainly focused on OAR and the work performed on prostate target classification has been limited.^22^, ^23^, ^27^ Syed et al.^23^ attempted to address target classification by defining a common non‐OAR class, but with no extended granularity. Sleeman Iv et al.^22^ expanded further and included the PTV for prostate gland but not the CTV or any other prostate RT targets. We believe the absence of general target classification to be an effect of the wide and challenging prostate target variety and the simultaneous existence of multiple GTVs, CTVs, and PTVs.

In our CT test data, we managed to classify 11 different targets with a minimum class individual F1 score of 96.2% (VesicleCTV). Rozario et al.^27^ used cleaned data, reported a 100% classification accuracy for their neural network and required only binary masks as input, similar to our model input. However, the model was only trained for five prostate organ classes, and the authors did not conduct any validation on independent test datasets. In the work of Yang et al.,^26^ a neural network model with seven prostate OAR classes was trained using transfer learning from their head and neck model. A recall of 100% was reported for five classes and a minimum of 81% for the other two.

In the work by Sleeman Iv et al.,^22^ a 95% and 90% F1 score was achieved on cleaned and uncleaned prostate data, respectively, highlighting the challenge of classifying real clinical uncleaned data. Our model could differentiate 22 label classes containing nine organs at risk, two support structures, one GTV target structure, and the CTV and PTV for five different targets with an unweighted classification accuracy of 98.8% and 99.8%, for uncleaned and cleaned CT test data (“other” class label removed), respectively. Corresponding weighted F1 scores were 98.4% and 99.8%. With respect to this, we believe our model has improved class diversity and usefulness for uncleaned real clinical data compared to previous publications and models.

Excellent results from prostate structure text label‐based methods have also been published by Schuler et al.^34^ with classification accuracy close to 100% and Syed et al.^23^ who reported unweighted F1 scores of 0.97 and 0.93 on internal and external test data. However, text‐based methods do not account for incorrect label names with respect to the geometric structure content^28^ or when the same label name is used for multiple structures.^23^ Interestingly, this problem was observed and manually corrected for a total of 290 times in our clinical training dataset prior to training, see section “creation of datasets.”

Further, during evaluation of the test data in our study, the model predicted five objects in the CT test dataset, one object in the MRI test dataset and four objects in the Umeå test dataset to a different class label than the suggested clinical ground truth. Upon detailed inspection, errors in the clinical ground truth label name were discovered. This did not alter the reported results in this study but demonstrated two important things: the major limitation of using text‐based methods for real clinical datasets and the powerful capabilities of our suggested method.

In the test CT dataset, 13 of 28 failing structures were mislabeled optimization structures, which demonstrate the importance of correctly labeled optimization structures as our method was not trained on such data. Extending training to include these class labels could be challenging as the structure contents can be very similar or identical to other targets or OAR structures. The classification performance on the MRI test dataset was slightly lower compared to the CT test dataset. In the MRI test dataset, six of the total seven failed structure classifications corresponded to CTV structures being classified as PTV (Figure 5, Table SA4). As double CTVs existed for each subject, where one of the CTVs was larger than the other CTV, similar to the geometric relation between CTV and PTV, it is probable that a CTV could be interpreted as a PTV, see Figure 2. However, the MRI dataset originated from a clinical study^39^ where the workflow differed compared to clinical routine. It is rather unlikely that clinical patient data will contain multiple CTVs or PTVs in such a manner. In any case, we believe that this can be accounted for by training the model with suitable data. A future improvement to avoid misinterpretations would be to use a combination of our model and existing target label text information, also noted in Syed et al.^23^ The QC method raised flags for body and couch structures for all subjects in the MRI data, and this was due to the smaller scan volume performed for MRI scanning compared to the CT‐based reference values. The results from the Umeå test dataset demonstrated the model's ability to generalize very well on trained class labels. However, 11 new class labels were found in the Umeå dataset compared to the training data, and this gave rise to a major part of failed classifications (Figure SA2.)

Misclassification due to lack of class label‐specific training data was a clear limitation in the method, as an assigned class label is determined from the largest class probability. This was demonstrated in the Umeå dataset, and the same applies for mislabeled optimization structures. The problem can be mitigated by including data for these labels or use boolean structure combinations as augmentation to enrich the training data. We also believe that a higher QC acceptance probability threshold could be of value to lower the false positives.

The QC method did not show a convincible performance for failed structure classifications as 55/88 QC flags in the CT test dataset were false positive and issued from structure volume analysis. Issuing warning with respect to structure volume might be a blunt tool due to the existing volume range overlap between many RT structures. As we release this software as open‐source, we hope to encourage future QC improvements and implementations of the method to other anatomical sites.

Future application of our method could enable automatic fusion, cleaning, and harmonization of multicenter datasets with differences in RT structure naming standards. This could further facilitate data‐driven machine learning research where intra‐ and interinstitutional collaborations compose the data source for clinical research. The same method could also be used to provide descriptive metadata and enrich existing clinical archive data. In the future, we aim to implement our model into the MICE toolkit (NONPI Medical AB, Sweden, Umeå) where the metadata can be supplied to an MIQA database, which provides structured unified storage of RT data at several Swedish University RT clinics.^30^

## 5 CONCLUSION

A DL‐based open‐source software for automatic renaming of prostate RT DICOM structure labels was developed and evaluated. The model was imaging modality independent and trained on multiple targets, OAR and support structures with state of the art classification accuracy in clinical CT, and MRI test datasets. Sufficient accuracy could be achieved with a moderate amount of training data, and the importance of input data representation was demonstrated. Clinic‐specific contouring standard however needs to be represented in the training data for successful use, and further improvements in QC assessment are needed.

## CONFLICT OF INTEREST

The authors declare that there is no conflict of interest that could be perceived as prejudicing the impartiality of the research reported.

## AUTHOR CONTRIBUTION

C. Jamtheim Gustafsson designed the study, led and participated in the data collection, and execution of the study. C. Jamtheim Gustafsson wrote the code for the method pipeline and carried out the analysis of the data. He is the main author of the work. M. Lempart participated in the data collection, design of the neural network, and the design of the neural network data input. He contributed substantially to result analysis and interpretation. J. Swärd participated in the design of the neural network and the design of the neural network data input. E. Persson provided expertise knowledge regarding clinical RT routines and interpretation of data for the work. T. Nyholm provided important intellectual content regarding expanding the method to contain prostate targets and not only organs at risk. T. Nyholm also provided input regarding future applications of the method. C. Thellenberg Karlsson collected and provided the test data set from Umeå (Sweden). J. Scherman gave expertise guidance regarding all machine learning parts of the study. He provided senior clinical expertise regarding the developed methodology and provided intellectual guidance in the result presentation, analysis, and interpretation. All authors revised and approved the final manuscript prior to submission.

## Supporting information

Supporting information
