# Supplementary material for: Deep learning‐based classification and structure name standardization for organ at risk and target delineations in prostate cancer radiotherapy
Source: J Appl Clin Med Phys. 2021 Oct 8;22(12):51–63. doi: 10.1002/acm2.13446 (PMC8664152; doi:10.1002/acm2.13446)
Supplement: Supplementary file 1 — Supporting information [file ACM2-22-51-s001.docx]

# Supplementary material

## Definition of classification metrics

The classification metrics precision, recall, F1 score and accuracy are used in this paper and are hereby defined and explained. The metrics are calculated from true positive (TP), false positive (FP), true negative (TN) and false negative (FN).

Precision: Calculated as TP/(TP+FP). Answers the question what proportion of total positive identifications was actually correct.

Recall (Sensitivity): Calculated as TP/(TP + FN). Answers the question what proportion of total actual positives was correctly identified.

F1 score: Calculated as 2 x Recall x Precision / (Recall + Precision). A weighted average of Precision and Recall.

Accuracy: Calculated as (TP+TN)/(TP+TN+FP+FN). Answers the question what the proportion of correct predations was compared to the total number of predictions.

**Table A1.** Clinically defined structure names in training data, CT test dataset and MRI test dataset together with description of the structure content, class label definition and number of available structures for each structure name and class label.

| Clinical RT structure name in training data | Description of RT structure name | Class label definition | Training data class label weight | # of RT structures with clinical RT structure name in training dataset (total in class label) | # of RT structures with clinical RT structure name in CT test dataset (total in class label) | # of RT structures with clinical RT structure name in MRI test dataset (total in class label) | Predefined inference RT structure name |
| --- | --- | --- | --- | --- | --- | --- | --- |
| BODY | Body | 0 | 0.53 | 1854 (1854) | 200 (200) | 40 (40) | BODY |
| Bladder | Bladder | 1 | 0.53 | 1849 (1849) | 200 (200) | 41 (41) | Bladder |
| FemoralHead_L | Left femoral head | 2 | 0.54 | 1804 (1804) | 195 (195) | 40 (40) | FemoralHead_L |
| FemoralHead_R | Right femoral head | 3 | 0.54 | 1801 (1801) | 194 (194) | 40 (40) | FemoralHead_R |
| Rectum | Rectum | 4 | 0.53 | 1850 (1850) | 202 (202) | 42 (42) | Rectum |
| Genitalia | Genitalia | 5 | 0.86 | 1198 (1136) | 135 (134) | 39 (39) | Genitalia |
| Penilebulb | Penile bulb | 6 | 0.88 | 1045 (1107) | 130 (131) | 38 (38) | PenileBulb |
| CouchSurface | Couch surface support structure | 7 | 0.53 | 1845 (1845) | 200 (200) | 40 (40) | CouchSurface |
| CouchInterior | Couch interior support structure | 8 | 0.53 | 1845 (1845) | 200 (200) | 40 (40) | CouchInterior |
| BowelBag | Bowel bag | 9 | 4.14 | 230 (230) | 23 (23) | 0 (0) | BowelBag |
| AnalCanal | Anal canal | 10 | 4.18 | 237 (237) | 21 (21) | 0 (0) | AnalCanal |
| CTVT1_780 | Prostate gland 78.0 Gy, 39 fractions | 11 | 0.70 | 652 (1391) | 78 (162) | 81 (81) | GlandCTV |
| CTVT1_427 | Prostate gland 42.7 Gy, 7 fractions | 11 |  | 4 | 1 | 0 | GlandCTV |
| CTVT_427 | Prostate gland 42.7 Gy, 7 fractions | 11 |  | 413 | 48 | 0 | GlandCTV |
| CTVT1_500 | Prostate gland 50.0 Gy, 25 fractions, combined with brachytherapy. | 11 |  | 72 | 9 | 0 | GlandCTV |
| CTVT_360 | Prostate gland 36.0 Gy, 6 fractions, palliative. | 11 |  | 0 | 0 | 0 | GlandCTV |
| CTVT1_510 | Prostate gland 51.0 Gy, 17 fractions, palliative. | 11 |  | 130 | 10 | 0 | GlandCTV |
| CTVT1_500pros | Prostate gland 50.0 Gy | 11 |  | 29 | 6 | 0 | GlandCTV |
| CTVT1_450 | Prostate gland 45.0 Gy, 15 fractions, palliative. | 11 |  | 91 | 6 | 0 | GlandCTV |
| PTVT1_780 | As above for respective CTV, 7 mm marginal | 12 | 0.61 | 656 (1594) | 75 (182) | 40 (40) | GlandPTV |
| PTVT1_427 | As above for respective CTV, 7 mm marginal | 12 |  | 10 | 2 | 0 | GlandPTV |
| PTVT_427 | As above for respective CTV, 7 mm marginal | 12 |  | 412 | 48 | 0 | GlandPTV |
| PTVT1_500 | As above for respective CTV, 7-10 mm marginal | 12 |  | 149 | 11 | 0 | GlandPTV |
| PTVT_360 | As above for respective CTV, 8 mm marginal posterior, 10 mm other directions. | 12 |  | 0 | 0 | 0 | GlandPTV |
| PTVT1_510 | As above for respective CTV, 7 mm marginal. | 12 |  | 130 | 10 | 0 | GlandPTV |
| PTVT1_500pros | As above for respective CTV, 7 or 10 mm marginal. | 12 |  | 143 | 20 | 0 | GlandPTV |
| PTVT1_450 | As above for respective CTV, 8 mm marginal posterior, 10 mm other directions. | 12 |  | 90 | 6 | 0 | GlandPTV |
| CTVT2_700 | Seminal vesicle 70.0 Gy, 35 fractions | 13 | 2.36 | 55 (409) | 6 (51) | 0 (0) | VesicleCTV |
| CTVT2_500 | Seminal vesicle 50.0 Gy, 25 fractions | 13 |  | 96 | 13 | 0 | VesicleCTV |
| CTVT3_500 | Seminal vesicle 50.0 Gy, 25 fractions | 13 |  | 29 | 1 | 0 | VesicleCTV |
| CTVT2_700ves | Seminal vesicle 70.0 Gy, 35 fractions | 13 |  | 14 | 3 | 0 | VesicleCTV |
| CTVT2_500ves | Seminal vesicle 50.0 Gy, 25 fractions | 13 |  | 110 | 12 | 0 | VesicleCTV |
| CTVT3_500ves | Seminal vesicle 50.0 Gy, 25 fractions | 13 |  | 9 | 1 | 0 | VesicleCTV |
| PTVT2_700 | As above for respective CTV, 10 mm marginal | 14 | 2.43 | 58 (397) | 7 (49) | 0 (0) | VesiclePTV |
| PTVT2_500 | As above for respective CTV, 10 mm marginal | 14 |  | 104 | 13 | 0 | VesiclePTV |
| PTVT3_500 | As above for respective CTV, 10 mm marginal | 14 |  | 32 | 1 | 0 | VesiclePTV |
| PTVT2_700ves | As above for respective CTV, 10 mm marginal | 14 |  | 14 | 3 | 0 | VesiclePTV |
| PTVT2_500ves | As above for respective CTV, 10 mm marginal | 14 |  | 106 | 12 | 0 | VesiclePTV |
| PTVT3_500ves | As above for respective CTV, 10 mm marginal | 14 |  | 9 | 1 | 0 | VesiclePTV |
| GTVN1_600 | Lymph nodes, 60.0 Gy, 25 fractions. | 15 | 6.75 | 117 (144) | 16 (21) | 0 (0) | IlLymGTV |
| GTVN2_600 | Lymph nodes, 60.0 Gy, 25 fractions. | 15 |  | 19 | 4 | 0 | IlLymGTV |
| GTVN3_600 | Lymph nodes, 60.0 Gy, 25 fractions. | 15 |  | 2 | 0 | 0 | IlLymGTV |
| GTVN1_640 | Lymph nodes, 64.0 Gy, 25 + 7 fractions. | 15 |  | 6 | 0 | 0 | IlLymGTV |
| CTVN1_600 | As above for respective GTV, 5 mm marginal | 16 | 6.60 | 115 (146) | 17 (21) | 0 (0) | IlLymCTV |
| CTVN2_600 | As above for respective GTV, 5 mm marginal | 16 |  | 19 | 4 | 0 | IlLymCTV |
| CTVN3_600 | As above for respective GTV, 5 mm marginal | 16 |  | 3 | 0 | 0 | IlLymCTV |
| CTVN1_640 | As above for respective GTV, 5 mm marginal | 16 |  | 7 | 0 | 0 | IlLymCTV |
| PTVN1_600 | As above for respective CTV, 5 mm marginal | 17 | 6.40 | 118 (150) | 16 (21) | 0 (0) | IlLymPTV |
| PTVN2_600 | As above for respective CTV, 5 mm marginal | 17 |  | 18 | 4 | 0 | IlLymPTV |
| PTVN3_600 | As above for respective CTV, 5 mm marginal | 17 |  | 3 | 0 | 0 | IlLymPTV |
| PTVN1_640 | As above for respective CTV, 5 mm marginal | 17 |  | 8 | 1 | 0 | IlLymPTV |
| CTVN_500 | Elective iliac lymph nodes, 50 Gy, 25 fractions | 18 | 2.51 | 385 (388) | 34 (38) | 0 (0) | IlElecCTV |
| PTVN_500 | As above for respective CTV, 8 mm marginal | 19 | 2.51 | 387 (388) | 35 (38) | 0 (0) | IlElecPTV |
| CTVT1_600 | Prostate bed with or without vesicle bed, 60.0 Gy, 30 fractions. | 20 | 2.31 | 0 (429) | 0 (42) | 0 (0) | BedCTV |
| CTVT1_700 | Prostate bed with or without vesicle bed, 70.0 Gy, 35 fractions. | 20 |  | 419 | 50 | 0 (0) | BedCTV |
| PTVT1_600 | As above for respective CTV, 10 mm marginal | 21 | 2.31 | 0 (429) | 0 (44) | 0 (0) | BedPTV |
| PTVT1_700 | As above for respective CTV, 10 mm marginal | 21 |  | 419 | 48 | 0 | BedPTV |

Observe that the total number of objects for each class label can be different from the sum of the class content. This is due to structure names not being present in the list or errors in the clinical data that were corrected, and hence the class label was also corrected. Class labels were used in the deep learning model training as the model was based on supervised learning. The class label weight, calculated from the class label frequency in the training data and used during training, was also included. The predefined inference structure name was assigned according to determined class label during model inference and was inserted into the new RT structure DICOM file.

**Table A2.** Classification performance per class label together with macro and weighted average for the whole CT test data (uncleaned data).

|  | Precision | Recall | F1 score | Number of structures |
| --- | --- | --- | --- | --- |
| AnalCanal | 1.0000 | 0.9524 | 0.9756 | 21 |
| BODY | 1.0000 | 1.0000 | 1.0000 | 200 |
| BedCTV | 0.9767 | 1.0000 | 0.9882 | 42 |
| BedPTV | 0.9565 | 1.0000 | 0.9778 | 44 |
| Bladder | 0.9900 | 0.9950 | 0.9925 | 200 |
| BowelBag | 0.8519 | 1.0000 | 0.9200 | 23 |
| CouchInterior | 1.0000 | 1.0000 | 1.0000 | 200 |
| CouchSurface | 1.0000 | 1.0000 | 1.0000 | 200 |
| FemoralHead_L | 0.9949 | 1.0000 | 0.9974 | 195 |
| FemoralHead_R | 0.9848 | 1.0000 | 0.9923 | 194 |
| Genitalia | 1.0000 | 1.0000 | 1.0000 | 134 |
| GlandCTV | 0.9877 | 0.9938 | 0.9908 | 162 |
| GlandPTV | 0.9836 | 0.9890 | 0.9863 | 182 |
| IlElecCTV | 1.0000 | 1.0000 | 1.0000 | 38 |
| IlElecPTV | 0.9500 | 1.0000 | 0.9744 | 38 |
| IlLymCTV | 0.9545 | 1.0000 | 0.9767 | 21 |
| IlLymGTV | 1.0000 | 0.9524 | 0.9756 | 21 |
| IlLymPTV | 1.0000 | 1.0000 | 1.0000 | 21 |
| Other | 0.0000 | 0.0000 | 0.0000 | 22 |
| PenileBulb | 1.0000 | 1.0000 | 1.0000 | 131 |
| Rectum | 0.9951 | 1.0000 | 0.9975 | 202 |
| VesicleCTV | 0.9273 | 1.0000 | 0.9623 | 51 |
| VesiclePTV | 0.9608 | 1.0000 | 0.9800 | 49 |
| macro avg | 0.9354 | 0.9514 | 0.9429 | 2391 |
| weighted avg | 0.9796 | 0.9883 | 0.9838 | 2391 |

**Table A3.** Classification performance per class label together with macro and weighted average for the whole CT test data (cleaned data).

|  | Precision | Recall | F1 score | Number of structures |
| --- | --- | --- | --- | --- |
| AnalCanal | 1.0000 | 0.9524 | 0.9756 | 21 |
| BODY | 1.0000 | 1.0000 | 1.0000 | 200 |
| BedCTV | 0.9767 | 1.0000 | 0.9882 | 42 |
| BedPTV | 0.9565 | 1.0000 | 0.9778 | 44 |
| Bladder | 1.0000 | 0.9950 | 0.9975 | 200 |
| BowelBag | 1.0000 | 1.0000 | 1.0000 | 23 |
| CouchInterior | 1.0000 | 1.0000 | 1.0000 | 200 |
| CouchSurface | 1.0000 | 1.0000 | 1.0000 | 200 |
| FemoralHead_L | 1.0000 | 1.0000 | 1.0000 | 195 |
| FemoralHead_R | 1.0000 | 1.0000 | 1.0000 | 194 |
| Genitalia | 1.0000 | 1.0000 | 1.0000 | 134 |
| GlandCTV | 0.9877 | 0.9938 | 0.9908 | 162 |
| GlandPTV | 1.0000 | 0.9890 | 0.9945 | 182 |
| IlElecCTV | 1.0000 | 1.0000 | 1.0000 | 38 |
| IlElecPTV | 1.0000 | 1.0000 | 1.0000 | 38 |
| IlLymCTV | 0.9545 | 1.0000 | 0.9767 | 21 |
| IlLymGTV | 1.0000 | 0.9524 | 0.9756 | 21 |
| IlLymPTV | 1.0000 | 1.0000 | 1.0000 | 21 |
| PenileBulb | 1.0000 | 1.0000 | 1.0000 | 131 |
| Rectum | 1.0000 | 1.0000 | 1.0000 | 202 |
| VesicleCTV | 1.0000 | 1.0000 | 1.0000 | 51 |
| VesiclePTV | 1.0000 | 1.0000 | 1.0000 | 49 |
| macro avg | 0.9943 | 0.9947 | 0.9944 | 2369 |
| weighted avg | 0.9975 | 0.9975 | 0.9975 | 2369 |

**Table A4.** Classification performance per class label together with macro and weighted average for the whole MRI test data (uncleaned data).

|  | Precision | Recall | F1 score | Number of structures |
| --- | --- | --- | --- | --- |
| BODY | 1.0000 | 1.0000 | 1.0000 | 40 |
| Bladder | 0.9762 | 1.0000 | 0.9880 | 41 |
| CouchInterior | 1.0000 | 1.0000 | 1.0000 | 40 |
| CouchSurface | 1.0000 | 1.0000 | 1.0000 | 40 |
| FemoralHead_L | 1.0000 | 1.0000 | 1.0000 | 40 |
| FemoralHead_R | 1.0000 | 1.0000 | 1.0000 | 40 |
| Genitalia | 1.0000 | 1.0000 | 1.0000 | 39 |
| GlandCTV | 1.0000 | 0.9259 | 0.9615 | 81 |
| GlandPTV | 0.8696 | 1.0000 | 0.9302 | 40 |
| Other | 0.0000 | 0.0000 | 0.0000 | 1 |
| PenileBulb | 1.0000 | 1.0000 | 1.0000 | 38 |
| Rectum | 1.0000 | 1.0000 | 1.0000 | 42 |
| macro avg | 0.9038 | 0.9105 | 0.9066 | 482 |
| weighted avg | 0.9851 | 0.9855 | 0.9846 | 482 |

**Table A5.** Classification performance per class label together with macro and weighted average for the whole MRI test data (cleaned data).

|  | Precision | Recall | F1 score | Number of structures |
| --- | --- | --- | --- | --- |
| BODY | 1.0000 | 1.0000 | 1.0000 | 40 |
| Bladder | 1.0000 | 1.0000 | 1.0000 | 41 |
| CouchInterior | 1.0000 | 1.0000 | 1.0000 | 40 |
| CouchSurface | 1.0000 | 1.0000 | 1.0000 | 40 |
| FemoralHead_L | 1.0000 | 1.0000 | 1.0000 | 40 |
| FemoralHead_R | 1.0000 | 1.0000 | 1.0000 | 40 |
| Genitalia | 1.0000 | 1.0000 | 1.0000 | 39 |
| GlandCTV | 1.0000 | 0.9259 | 0.9615 | 81 |
| GlandPTV | 0.8696 | 1.0000 | 0.9302 | 40 |
| PenileBulb | 1.0000 | 1.0000 | 1.0000 | 38 |
| Rectum | 1.0000 | 1.0000 | 1.0000 | 42 |
| macro avg | 0.9881 | 0.9933 | 0.9902 | 481 |
| weighted avg | 0.9892 | 0.9875 | 0.9877 | 481 |

**Table A6.** Classification performance per class label together with macro and weighted average for the whole Umeå test data (uncleaned data).

|  | Precision | Recall | F1 score | Number of structures |
| --- | --- | --- | --- | --- |
| AnalCanal | 0.9885 | 0.9773 | 0.9829 | 88 |
| BODY | 0.8929 | 1.0000 | 0.9434 | 100 |
| BedCTV | 0.5000 | 1.0000 | 0.6667 | 1 |
| BedPTV | 0.5000 | 1.0000 | 0.6667 | 1 |
| Bladder | 1.0000 | 0.9500 | 0.9744 | 100 |
| BoostGlandGTV | 0.0000 | 0.0000 | 0.0000 | 23 |
| BoostGlandPTV | 0.0000 | 0.0000 | 0.0000 | 21 |
| BowelBag | 0.2500 | 1.0000 | 0.4000 | 16 |
| CouchBolus | 0.0000 | 0.0000 | 0.0000 | 47 |
| CouchSurface | 0.0000 | 0.0000 | 0.0000 | 0 |
| FemoralHead_L | 0.9897 | 1.0000 | 0.9948 | 96 |
| FemoralHead_R | 0.9796 | 1.0000 | 0.9897 | 96 |
| Genitalia | 0.0000 | 0.0000 | 0.0000 | 0 |
| Gland+IlElec+VesicleCTV | 0.0000 | 0.0000 | 0.0000 | 12 |
| Gland+IlElec+VesiclePTV | 0.0000 | 0.0000 | 0.0000 | 35 |
| Gland+VesicleCTV | 0.0000 | 0.0000 | 0.0000 | 7 |
| Gland+VesiclePTV | 0.0000 | 0.0000 | 0.0000 | 1 |
| GlandCTV | 0.8348 | 0.9697 | 0.8972 | 99 |
| GlandPTV | 0.9018 | 1.0000 | 0.9484 | 101 |
| IlElec+VesicleCTV | 0.0000 | 0.0000 | 0.0000 | 17 |
| IlElec+VesiclePTV | 0.0000 | 0.0000 | 0.0000 | 1 |
| IlElecCTV | 0.4314 | 0.9565 | 0.5946 | 23 |
| IlElecPTV | 0.0256 | 1.0000 | 0.0500 | 1 |
| IlLymCTV | 0.0000 | 0.0000 | 0.0000 | 0 |
| IlLymGTV | 0.1887 | 1.0000 | 0.3175 | 10 |
| IlLymPTV | 1.0000 | 0.6000 | 0.7500 | 10 |
| Other | 0.0000 | 0.0000 | 0.0000 | 11 |
| PenileBulb | 0.8305 | 1.0000 | 0.9074 | 98 |
| Rectum | 0.9900 | 1.0000 | 0.9950 | 99 |
| SmallBowel | 0.0000 | 0.0000 | 0.0000 | 18 |
| Urethra | 0.0000 | 0.0000 | 0.0000 | 34 |
| VesicleCTV | 0.9500 | 1.0000 | 0.9744 | 19 |
| VesiclePTV | 0.5000 | 1.0000 | 0.6667 | 1 |
| macro avg | 0.3865 | 0.5289 | 0.4157 | 1186 |
| weighted avg | 0.7284 | 0.7960 | 0.7522 | 1186 |

**Table A7.** Classification performance per class label together with macro and weighted average for the whole Umeå test data (cleaned data).

|  | Precision | Recall | F1 score | Number of structures |
| --- | --- | --- | --- | --- |
| AnalCanal | 1.0000 | 0.9773 | 0.9885 | 88 |
| BODY | 1.0000 | 1.0000 | 1.0000 | 100 |
| BedCTV | 0.5000 | 1.0000 | 0.6667 | 1 |
| BedPTV | 0.5000 | 1.0000 | 0.6667 | 1 |
| Bladder | 1.0000 | 0.9500 | 0.9744 | 100 |
| BowelBag | 1.0000 | 1.0000 | 1.0000 | 16 |
| FemoralHead_L | 1.0000 | 1.0000 | 1.0000 | 96 |
| FemoralHead_R | 1.0000 | 1.0000 | 1.0000 | 96 |
| Genitalia | 0.0000 | 0.0000 | 0.0000 | 0 |
| GlandCTV | 1.0000 | 0.9697 | 0.9846 | 99 |
| GlandPTV | 0.9352 | 1.0000 | 0.9665 | 101 |
| IlElecCTV | 1.0000 | 0.9565 | 0.9778 | 23 |
| IlElecPTV | 0.5000 | 1.0000 | 0.6667 | 1 |
| IlLymCTV | 0.0000 | 0.0000 | 0.0000 | 0 |
| IlLymGTV | 1.0000 | 1.0000 | 1.0000 | 10 |
| IlLymPTV | 1.0000 | 0.6000 | 0.7500 | 10 |
| PenileBulb | 1.0000 | 1.0000 | 1.0000 | 98 |
| Rectum | 1.0000 | 1.0000 | 1.0000 | 99 |
| VesicleCTV | 1.0000 | 1.0000 | 1.0000 | 19 |
| VesiclePTV | 1.0000 | 1.0000 | 1.0000 | 1 |
| macro avg | 0.8218 | 0.8727 | 0.8321 | 959 |
| weighted avg | 0.9916 | 0.9844 | 0.9870 | 959 |


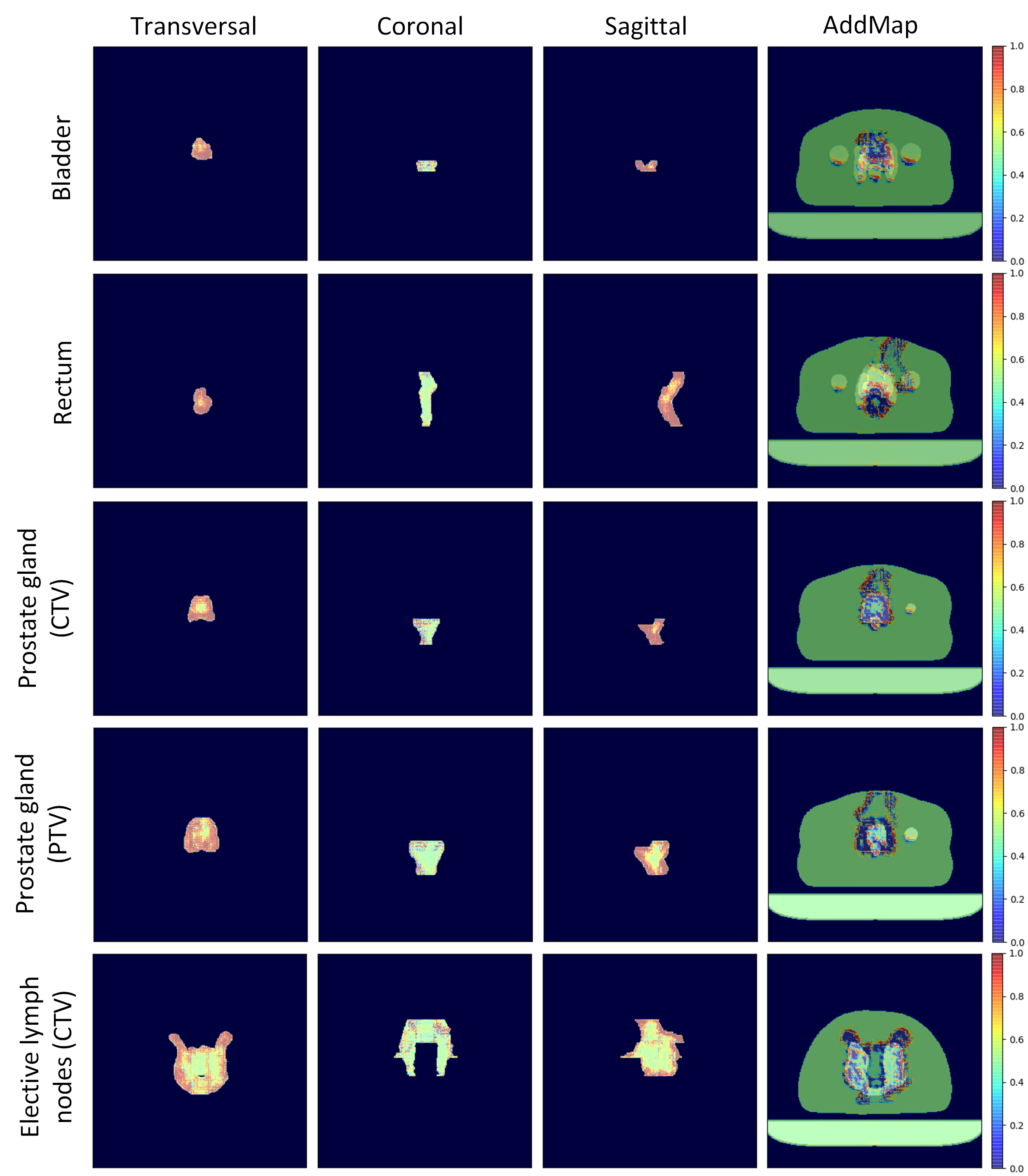


**Fig.A1**. Input images to the model as projection data for transversal, coronal and sagittal views together with corresponding AddMap for the described organs at risks and targets (same subject as figure 2 in the paper). Masked Guided Grad-Cam saliency maps are overlaid on the structures and the most influential pixels for determining the respective class label are shown as high values (maximum 1) and color coded. Some signal leakage between the channels/images can be observed as the input data are being combined after the first convolutional layer in the network. Images have been rotated and/or flipped for improved viewing. The valuable information from AddMap, used in CTV and PTV differentiation on row 3 and 4, can be observed in the AddMap of prostate gland CTV and PTV, see also Fig.2. in the paper for an extensive explanation.


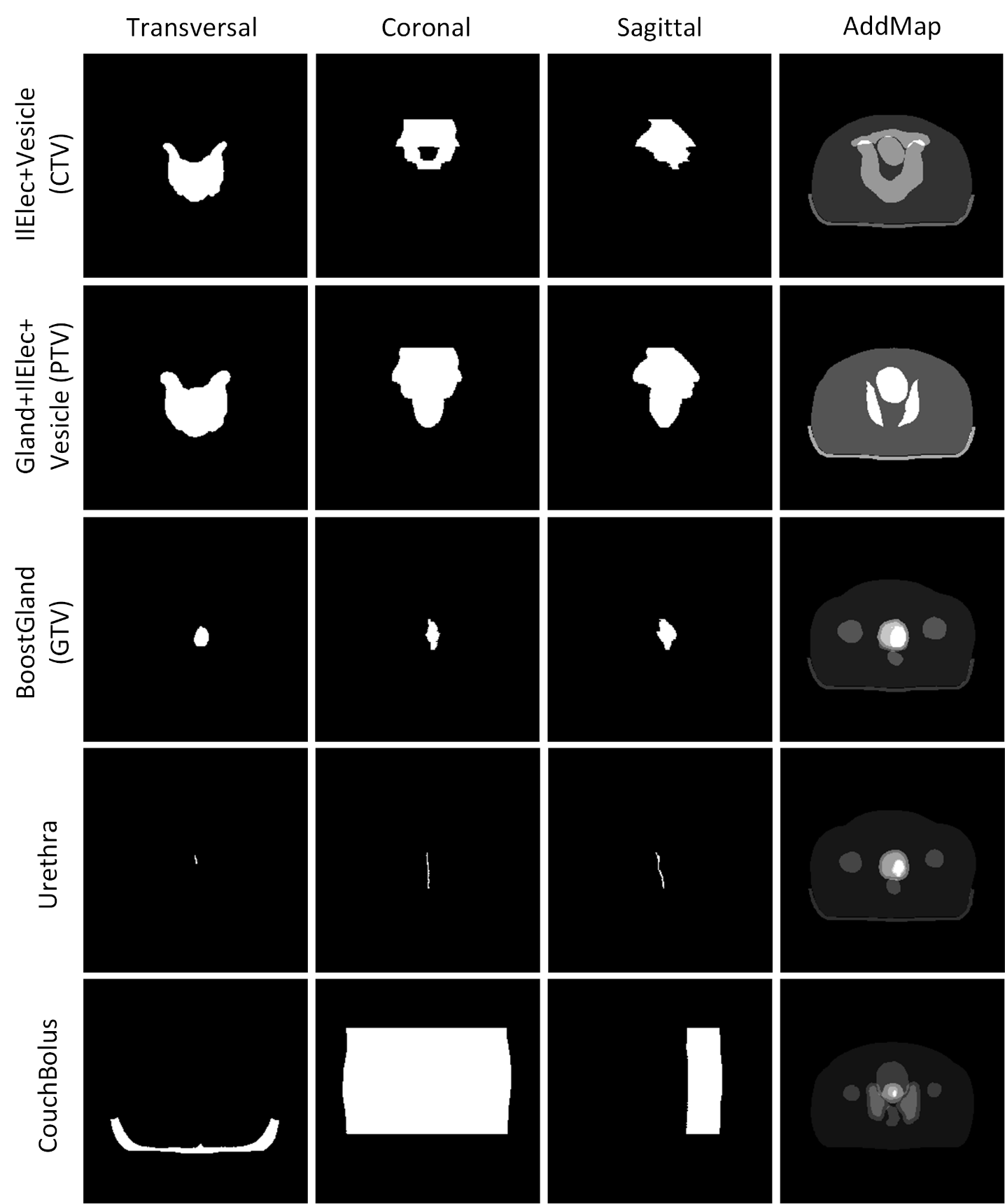


**Fig.A2**. Five examples of new class labels from the Umeå test dataset on which the model was not trained, presented as projection data for transversal, coronal, and sagittal views together with corresponding AddMap. Unlike the data from Skåne University Hospital, the CTV and PTV target volumes included multiple structures in a variety of boolean combinations. An elective lymph node structure combined with the vesicle is shown in row 1 as a CTV. The target PTV structure on row 2 is however based on a fusion of the structure in row 1 and the prostate gland (not shown). The visual difference in the projection data due to boolean operations can be seen when comparing it to an isolated elective lymph node CTV, such as the one in in fig.2. The difference is clearly pronounced in the coronal projection. The prostate gland GTV boost volume is shown in row 3 and the urethra, defined as an organ at risk, at row 4. The CouchBolus is defined as a support structure, shown in row 5. It is used in the Umeå radiotherapy clinic as the treatment planning system cannot by itself create a virtual treatment couch. Classification results for these class labels can be found in Fig.6. and Table A6. Images originate from the same patient and have been rotated and/or flipped for improved viewing. Individual gray scales were applied to AddMap in the figure to visualize each organ, actual pixel values were assigned in the same way for all individual structure AddMap.
